# Uncovering the Metabolic Strategies of the Dormant Microbial Majority: towards Integrative Approaches

**DOI:** 10.1128/mSystems.00107-19

**Published:** 2019-05-14

**Authors:** Chris Greening, Rhys Grinter, Eleonora Chiri

**Affiliations:** a School of Biological Sciences, Monash University, Clayton, Victoria, Australia

**Keywords:** hydrogen, metabolism, mycobacteria, persistence, soil microbiology, trace gases

## Abstract

A grand challenge in microbiology is to understand how the dormant majority lives. In natural environments, most microorganisms are not growing and instead exist in a spectrum of dormant states. Despite this, most research on microbial metabolism continues to be growth-centric, and many overlook the fact that dormant cells require energy for maintenance. In this perspective, we discuss our research program to uncover the metabolic strategies that support microbial survival. We present two major principles underlying these studies. The first is the recent realization that microbial survival depends on previously unrecognized metabolic flexibility. The second is that new discoveries in this area depend on more sophisticated integration of approaches at the molecular, cellular, and ecosystem levels. These principles are illustrated with examples from the literature, including our own work demonstrating that bacteria can live on air, and areas for future methodological and theoretical development are highlighted.

## Abstract (precis)

A grand challenge in microbiology is to understand how the dormant majority lives. In natural environments, most microorganisms are not growing and instead exist in a spectrum of dormant states.

## PERSPECTIVE

It is well recognized that most microorganisms in natural environments exist in dormant rather than growing states (1). Yet, microbiology continues to be an overwhelmingly growth-centric field. This partly reflects methodological limitations: culture-dependent approaches are skewed toward relatively fast growers and culture-independent approaches often focus on the abundant few. These issues have been compounded by misconceptions about the nature and role of dormant microorganisms in different ecosystems. Many incorrectly synonymize dormancy with inactivity. In fact, dormant cells still maintain a low level of metabolic activity to maintain basal cell functions (1). Moreover, it is commonly thought that growing microorganisms are the most important players in natural ecosystems. However, many critical ecosystem services such as atmospheric gas regulation (2), antibiotic production (3), and even the maintenance of biodiversity itself (4) are mediated by dormant microorganisms.

The goal of our laboratory is to reshape understanding of how microorganisms persist in different environments. To achieve this, we combine culture-dependent and culture-independent approaches to investigate how microorganisms adapt their metabolism when limited for the electron donors and acceptors required for growth. The central theory driving this research is that microbial survival depends on previously unrecognized metabolic flexibility. To demonstrate this, we have developed an ambitious interdisciplinary research program that aims to (i) identify novel metabolic processes, (ii) resolve their molecular basis, and (iii) understand their ecological significance. In turn, we are applying these findings to address specific questions about global change, infectious disease, and biodiversity. This perspective will summarize the concepts and methodologies driving the development of this research program, while highlighting areas and opportunities for further development in this research space.

## MICROORGANISMS RELY ON HIDDEN METABOLIC FLEXIBILITY TO PERSIST

There is growing evidence that the growth-centric studies have underestimated the metabolic capabilities of microorganisms. It is often thought that, during dormancy, bacteria survive primarily by downregulating the processes they use for growth. However, we have observed that aerobic bacteria often broaden their metabolic repertoire during persistence. For example, obligate heterotrophs scavenge inorganic energy sources during carbon starvation (2, 5), obligate aerobes use fermentation as a last resort during hypoxia (6), and methanotrophs will take advantage of hydrogen (H_2_) whenever available (7). Thus, the traditional metabolic classifications used in systematic bacteriology break down when the focus is shifted from growth to survival. Other groups have also revealed surprising metabolic flexibility of lithotrophic aerobic bacteria from a wide range of environments (8–10).

An example of the discrepancies caused by growth-centric microbiology research is the area of atmospheric H_2_ oxidation. It was discovered in the 1970s that soils consume large amounts of atmospheric H_2_ and serve as the major biogeochemical sink of this gas. However, it wasn’t until over three decades later that the bacteria and enzymes responsible were finally identified, through efforts led by Constant and Conrad (2, 11, 12). This delay reflects that previous cultivation efforts were focused on isolating chemolithoautotrophs, i.e., bacteria that grow on H_2_ as the sole energy source. However, the bacteria mediating atmospheric uptake in fact grow heterotrophically and switch to using H_2_ during dormancy. Several long-known isolates, including Mycobacterium smegmatis (2), Streptomyces avermitilis (13), and Thermomicrobium roseum (14), have since been shown to also persist on atmospheric H_2_. Since this breakthrough, our genetic and biochemical studies have shown this process is mediated by specialized high-affinity, oxygen-tolerant [NiFe]-hydrogenases that input electrons from atmospheric H_2_ oxidation into the aerobic respiratory chain (2). Deletion of these enzymes results in reduced long-term survival during starvation and hypoxia (2, 6, 13). It was ultimately the growth-centric perspective on the microbial world, rather than methodological limitations, that prevented these biogeochemically and ecologically important discoveries from being made earlier.

It is now increasingly realized that atmospheric trace gases are hidden energy sources supporting the dormant microbial majority in aerated ecosystems. Genomic surveys have shown that isolates from the nine most dominant soil phyla also encode the key enzymes for aerobic H_2_ oxidation (15), and three such phyla have now been experimentally shown to scavenge H_2_ (5, 11, 14). In parallel, metagenomic studies revealed that high-affinity hydrogenases are highly abundant in soil ecosystems (15, 16). Particularly important is the discovery that atmospheric trace gases are the main energy sources supporting communities in some of the most hostile environments of all: Antarctic dry deserts (16). Whereas it was conventionally thought that oxygenic photosynthesis drives primary production in such ecosystems, physicochemical pressures such as water limitation generally exclude phototrophs from these soils. Instead, the dominant community members of such ecosystems are actually gas-scavenging bacteria, and biogeochemical assays show they mediate rapid levels of atmospheric H_2_ oxidation (16). Thus, in this case, findings from pure culture about how bacteria survive carbon starvation were also extendable to the ecosystem scale.

Resource generalism provides a competitive advantage for bacteria in deprived or changeable environments. Contrary to the classical paradigms of diauxic growth, bacteria may be more competitive if they continually cometabolize a broad range of energy sources. Atmospheric trace gases may be particularly desirable: while insufficiently concentrated to support growth, they are dependable energy sources for persistence given their ubiquity and diffusibility. Metabolic flexibility may also be important for enabling organisms to maintain energy needs in highly changeable environments. For example, in a further extension of our pure culture findings (6), we have shown that facultative fermentation is critical for the stability and productivity of the predominantly aerobic communities within dynamic intertidal sand sediments (17). Going forward, we are using similar philosophies to address key questions about a range of environmentally and medically important microorganisms. For example, how does metabolic flexibility influence the microorganisms that control the production and consumption of greenhouse gases? How do bacterial pathogens remodel their metabolism to sustain energy needs in different environmental and host reservoirs?

## INTEGRATIVE APPROACHES ENABLE DISCOVERY AND CHARACTERIZATION OF PERSISTENCE MECHANISMS

Despite considerable progress, much more research is needed to gain a systematic understanding of how most microorganisms persist in different environments. Through rapid developments in meta-omics technologies, researchers have developed an increasingly comprehensive knowledge of the phylogenetic diversity and functional potential of microorganisms in different biomes. However, these technologies can provide only inferences about how these organisms live, rather than the sorts of proofs derived from genetic, biochemical, or physiological approaches. Moreover, given that these technologies primarily rely on findings about genes carried by cultivated species, they have limited capacity to discover or characterize genes encoding novel function; reflecting this, over half of the genes detected in candidate phyla remain completely functionally unannotated (18). We argue that improved understanding of microbial metabolism depends on integrating meta-omics technologies with more targeted approaches. Only through such combinations will it be possible to gain both a broad and a deep understanding of how the uncultured majority live.

Reflecting this, many of the greatest metabolic discoveries in the last few years have depended on synergies between meta-omic and targeted approaches. For example, following the discovery of the first complete ammonia-oxidizing (comammox) bacteria through genome-resolved metagenomics (19, 20), the first isolate of such an organism was cultured (Nitrospira inopinata); this provided a more sophisticated understanding of their ecophysiology, revealing that comammox bacteria are high-affinity ammonia oxidizers adapted for an oligotrophic lifestyle (21). Innovations are also possible independent of culturing. For example, metagenomic approaches led to the discovery of novel archaeal lineages harboring divergent methyl-CoM reductase genes (22); the subsequent experimental detection of the unprecedented intermediate butyl-coenzyme M in a butane-oxidizing enrichment led to the realization that some such enzymes mediate unprecedented pathways of anaerobic alkane activation (23). In addition, protein biochemistry has also been used on rare occasions to reveal information about otherwise intractable processes. In a particularly impressive example, Krüger et al. confirmed how anaerobic methane oxidation occurs by directly purifying the key enzyme responsible from a methane-oxidizing microbial mat (24); this supported a weight of evidence from metagenomic, biogeochemical, and imaging studies that anaerobic methanotrophs (ANME) function through performing a reverse methanogenesis pathway.

**FIG 1:**
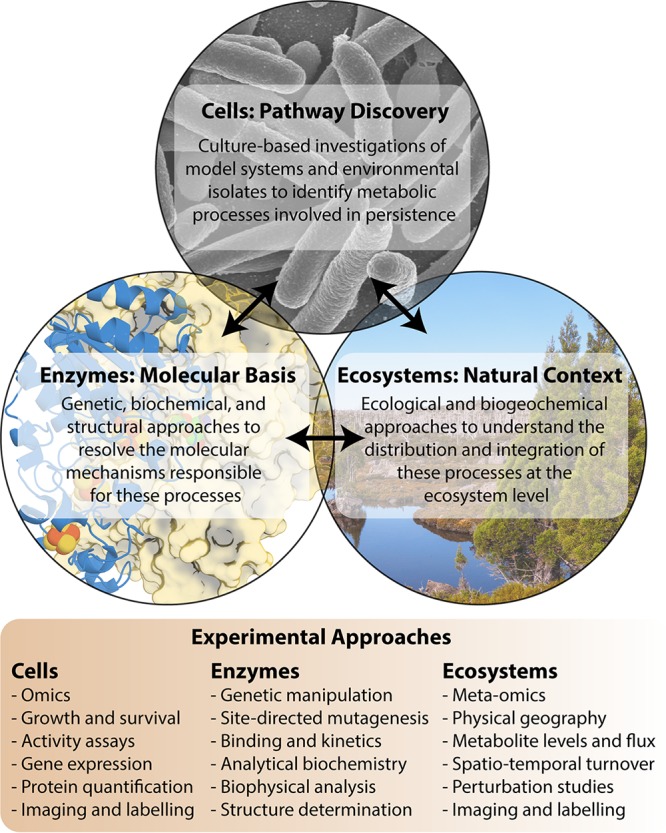
Overview of how synthesizing information across enzymatic, cellular, and ecosystem scales enables the discovery and characterization of metabolic processes involved in microbial persistence.

In our experience, integrative approaches are rewarding because of the different perspectives they provide and the synergies that can be achieved by working across disciplines. As outlined in Fig. 1, our own research program is becoming highly integrated. We benefit from diverse expertise, both within our group and through various collaborations, to develop information about survival-related processes at the protein, cellular, and ecosystem levels. We then integrate these findings to gain a comprehensive understanding of the basis and significance of different metabolic processes. For example, through such approaches, we are currently developing understanding of how atmospheric H_2_ oxidation is mediated at both the molecular level and the ecosystem scale. These approaches are largely inspired by our experiences in medical microbiology, where integrative approaches are commonplace. For example, similar approaches have transformed understanding of how pathogens such as Mycobacterium tuberculosis generate energy during latent infection of host tissues; a gold standard approach is to identify novel metabolic enzymes through systems biology approaches, determine their function and mechanism through biochemical and genetic approaches, and validate their importance through phenotypic studies both *in vitro* and *in vivo* (25). There are also some areas of environmental microbiology where strong enzyme-to-ecosystem links have also been developed, for example, in the area of microbial methane cycling (26), but these are presently exceptions rather than the rule.

Looking ahead, several ongoing methodological developments have the potential to transform knowledge of how the microbial majority live. First, a range of labeling and imaging approaches enable researchers to precisely determine which microorganisms mediate certain biogeochemical processes. For example, highly resolved mass spectrometry techniques such as nanoscale secondary-ion mass spectrometry (NanoSIMS) allow detection of specific metabolic activities at the single-cell level. In parallel, the development of high-throughput culturing approaches (also known as culturomics) facilitates the bacteriological and genomic description of new isolates at unprecedented scale. In our view, these approaches ought to be combined with in-depth approaches to gain a sophisticated understanding of the ecophysiology of organisms, for example, through profiling gene expression and enzymatic activity of cells in their dormant states. We also predict that genetic and biochemical approaches will become more broadly available for a range of systems. Currently, genetic tools are unavailable for many ecologically dominant and biogeochemically important phyla, including *Acidobacteria*, *Thaumarchaeota*, *Nitrospirae*, and *Verrucomicrobia*; however, recent developments in CRISPR interference technologies may start to change this (27). Finally, from a biochemistry standpoint, the rise of cryo-electron microscopy has allowed for protein structure determination at high resolution, using extremely small volumes of purified sample. This has allowed for structure determination of low-copy-number proteins expressed in their native organism, ushering in a new chapter of structural biology (28). We argue that, by leveraging both current and emerging technologies, it will be possible for researchers to gain a broad and deep understanding of the dormant majority.
